# Giant right ventricular outflow tract thrombus in hereditary spherocytosis: a case report

**DOI:** 10.1186/s12959-016-0083-3

**Published:** 2016-04-26

**Authors:** Cedric Davidsen, Terje Hjalmar Larsen, Eva Gerdts, Mai Tone Lønnebakken

**Affiliations:** Department of Heart Disease, Haukeland University Hospital, Bergen, Norway; Department of Clinical Science, University of Bergen, Bergen, Norway

**Keywords:** Thrombus, Right ventricle, Contrast echocardiography, Hereditary spherocytosis, Pulmonary embolism

## Abstract

**Background:**

In hereditary spherocytosis with severe anemia, splenectomy is a recommended treatment. However, the spleen carries an important role both in immune function and coagulation. The increased risk of bacterial infections associated with splenectomy is well known. Recently, hypercoagulation disorders have also been linked to splenectomy through loss of regulation of platelet activity, loss of filtering function and post-splenectomy thrombocytosis.

**Case presentation:**

A 28 year-old smoking women who had previously undergone splenectomy due to hereditary spherocytosis with a moderate thrombocytosis (platelet count 553–635*10^9^/L), presented with recurrent episodes of pulmonary embolisms. Further examination by multimodality cardiac imaging demonstrated a giant chronic thrombus in the right ventricular outflow tract, which eventually had to be surgically removed.

**Conclusions:**

The present case highlights the increased risk of severe thromboembolic complications following therapeutic splenectomy in hereditary spherocytosis, and emphasis the important role of multimodality cardiac imaging in recurrent pulmonary embolism, diagnosing a giant chronic thrombus in the right ventricular outflow tract.

**Electronic supplementary material:**

The online version of this article (doi:10.1186/s12959-016-0083-3) contains supplementary material, which is available to authorized users.

## Background

Hereditary spherocytosis is a genetic disease affecting membrane proteins of the erythrocytes responsible for interactions with the cytoskeleton. This leads to loss of the biconcave disk shape and results in spheric erythrocytes. These abnormal erythrocytes are removed by the spleen leading to various degrees of hemolytic anemia [1]. In patients with severe hemolytic anemia, splenectomy is very effective in reducing hemolysis, but it is associated with an increased risk of infections, particularly those caused by encapsulated bacteria such as Streptococcus pneumoniae and Haemophilus influenza [2]. In addition to bacterial infections, recent publications suggest a link between splenectomy and thromboembolic complications in patients with spherocytosis [3–9]. In this case report, we present a young splenectomized women with hereditary spherocytosis complicated by recurrent pulmonary embolism due to a chronic right ventricular outflow tract thrombus, which eventually was surgically removed.

## Case presentation

A 28 year old woman with hereditary spherocytosis who had undergone splenectomy at the age of 11 was admitted to a local hospital with hemoptysis and abdominal pain. The diagnosis hereditary spherocytosis, was based on family history, anemia and detection of spherocytes, the diagnosis has not been re-confirmed by eosin-5-maleimide (EMA) binding test in the recent past, and no mutation testing has been performed. Blood tests revealed leukocytosis (White blood cell count 18.5-16.8*10^9^/L), normal C-reactive protein and elevated D-dimer. The hemoglobin level was normal (14.6–15.0 g/dL), but spherocytes was noticed in the blood smear. A mild thrombocytosis (platelet count during hospital stays differed between 553–635*10^9^/L) was noticed. Due to the elevated D-dimer a helical computer tomography (CT) pulmonary angiography was performed, which revealed no pulmonary embolism, but a lung consolidation in the lateral part of the left inferior lobe, interpreted as pneumonia, and the patient was treated with antibiotics.

**Fig. 1:**
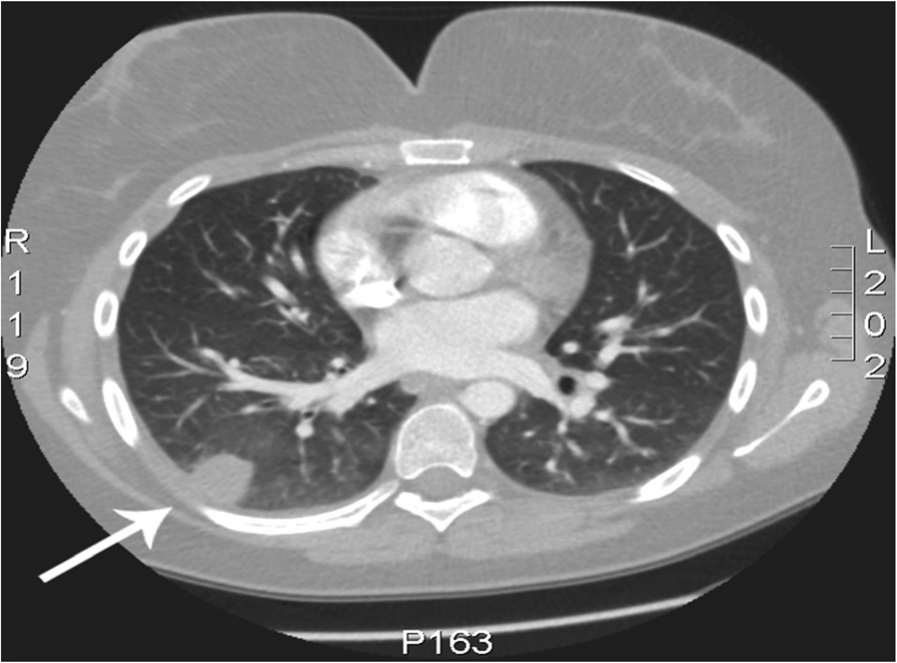
Thoracic CT-scan showing a consolidation in the right inferior lobe (arrow) interpreted as a pulmonary infarction due to pulmonary embolism from the cardiac thrombus

**Fig. 2:**
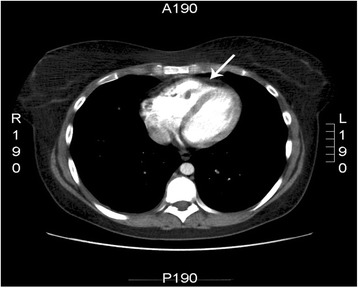
CT-pulmonary angiography that aroused suspicion of a thrombus in the right ventricle (arrow)

A year later the patients was re-admitted for a new episode of hemoptysis. A second helical CT-pulmonary angiography was performed, showing a consolidation in the right inferior lobe (Fig. 1) and suspicion of a thrombus in the right ventricle (Fig. 2). The lung consolidation was interpreted as a pulmonary infarction due to pulmonary embolism from the cardiac thrombus. Consequently, anticoagulation therapy with low-molecular weight heparin followed by warfarin was initiated. Apart from being a smoker, the medical history did not identify any classic risk factor for pulmonary embolism. In particularly, no family history of thrombosis, obesity, use of contraceptives, immobilization or recent long flights, injury or surgery associated with increased risk of thrombosis was present. No thrombophilic disorders (protein S deficiency, protein C deficiency, Factor V Leiden/activated protein C resistance, antithrombin (AT III) deficiency) were identified on blood tests. Antiphospholipid antibodies, lupus anticoagulant, anti-kardiolipin and anti-beta2-glycoprotein 1 were all negative, homocystein analysis was not performed. No clinical sign of inflammatory or malignant disease was identified during the diagnostic work-up, except for å mild leukocytosis and thrombocytosis that was regarded to be part of the thrombotic disease and previous splenectomy. A supplemental bone marrow biopsy to rule out any myelodysplastic syndrome was not performed as the patient was unwilling.

**Fig. 3:**
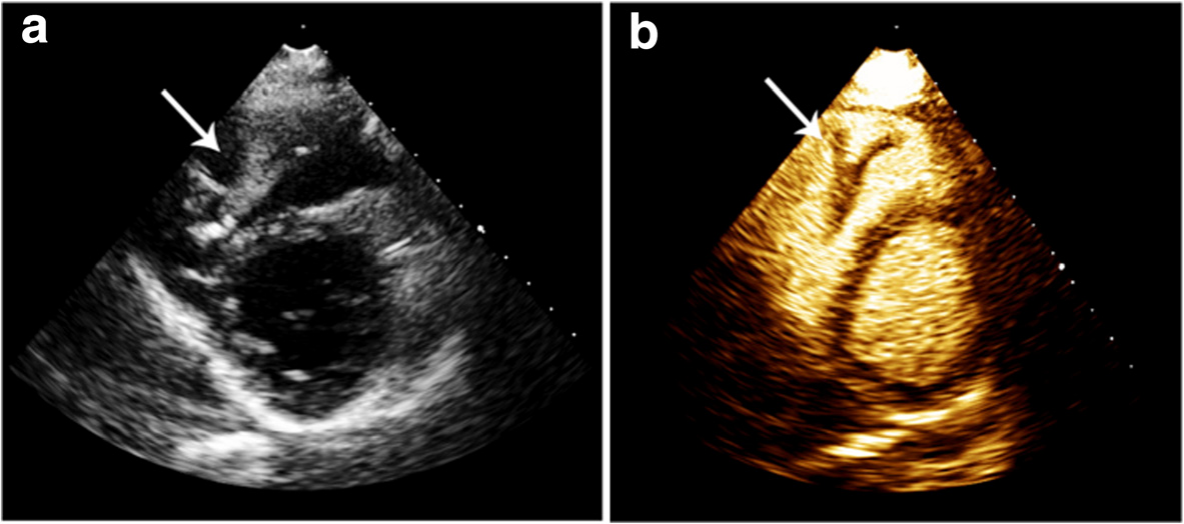
Transthoracic echocardiography showing a giant right intra-ventricular structure (arrow) in a parasternal short axis view (Panel **a**). The characteristic lack of contrast enhancement in the right intra-ventricular cavity confirms the presence of a giant right ventricular thrombus (Panel **b**)

**Fig. 4:**
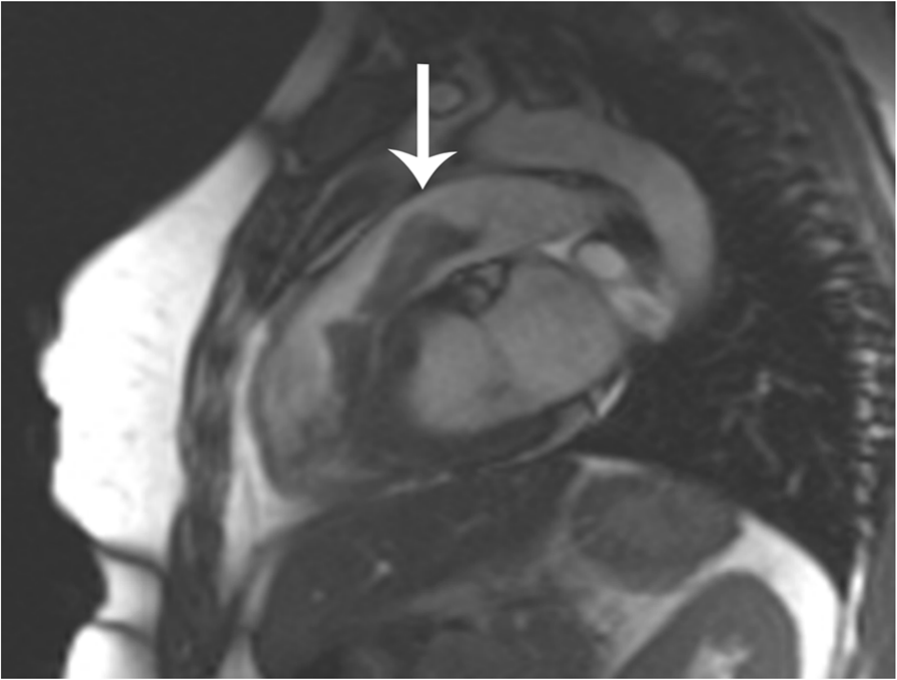
Cardiac magnetic resonance imaging in a sagittal plane demonstrating the piston-like shape of the thrombus, and how it protrudes through the pulmonary valve in systole

After 6 months of continuous anticoagulation therapy, the patient was readmitted for the third time due to shortness of breath and hemoptysis. A new CT pulmonary angiography showed multiple pulmonary embolisms and a persistent structure in the right ventricle. Multimodality imaging of the intracavitary cardiac structure was then performed. Transthoracic echocardiography revealed an elongated highly mobile structure attached to the right ventricular septum extending through the right ventricle outflow tract and into the pulmonary trunk (Fig. 3, panel a, Additional file 1). Supplemental contrast-enhanced imaging revealed no contrast uptake in the structure, consistent with a large right ventricular thrombus (Fig. 3, panel b, Additional file 2). Cardiac magnetic resonance imaging (MRI) confirmed an approximately 7 cm long thrombus attached to the apical part of the right ventricular septum protruding through the pulmonary valve in systole, but retracting to just below the pulmonary valve in diastole (Fig. 4, Additional file 3). Due to persistence of the thrombus and recurrent pulmonary embolism despite adequate anticoagulation therapy, surgical removal was performed, and the thromboembolic nature of the excised structure was confirmed by pathological examination. Postoperatively, long-term treatment with low- molecular - weight heparin injections was recommended. Echocardiography 6 months postoperatively was normal.

Recently, it has been acknowledged that splenectomy in hereditary spherocytosis is associated with a significant increased long-term risk of venous thromboembolic events, and severe chronic thromboembolic pulmonary hypertension has been reported in patients with hereditary spherocytosis after splenectomy [6, 7, 9, 10]. The current case adds to these by demonstrating a giant thrombus in the right ventricle as source for recurrent pulmonary infarctions despite adequate anticoagulation therapy, in a patient with hereditary spherocytosis and previous splenectomy, in which smoking was the only classic risk factor for increased thrombosis risk detected during the diagnostic work-up. A possible mechanism for the observed thrombosis risk after splenectomy is loss of splenic regulation of platelet activity and clearing of platelets from the bloodstream resulting in a post-splenectomy thrombocytosis and the loss of filter function in the spleen leading to an accumulation of pro-thrombotic blood elements [11]. The present case underlines that the benefit of splenectomy in reducing hemolysis in hereditary spherocytosis should be weighed against the increased long-term risk of severe thromboembolism and severe infections [12]. More recently, partial splenectomy is emerging as a possible treatment option to overcome these complications, although long-term data on infection and vascular risk are still lacking [13].

The impact of splenectomy on the increased thromboembolic risk was demonstrated in a prospective observational study of 634 patients from families with hereditary spherocytosis, followed from 1960 to 2007, finding patients who underwent splenectomy to have a 3-fold increased rate of thrombotic events compared to those who did not [8]. Nevertheless, current guidelines do not recommend extended thrombosis prophylaxis after splenectomy in patients with hereditary spherocytosis [1]. In our patient, apart from being a smoker, no other precipitating cause for pulmonary embolism was identified, and thus we believe it was caused by the splenectomy-associated risk for thrombosis. In an experimental study in mice splenectomy was associated with larger and more persistent thrombi and delayed thrombus resolution during anticoagulation therapy, paralleling the findings in our clinical case [11].

Right ventricular thrombi are present in approximately 4 % of patients with pulmonary embolisms, and are associated with a high mortality [14]. Although the current ESC guidelines on pulmonary embolism [15] does not warrant routine echocardiography in clinically stable patients with pulmonary embolism, repeated pulmonary embolisms and a suspected right ventricular thrombus on CT pulmonary angiography at the second hospitalization should have prompted additional cardiac imaging at an earlier point of time in our case and possibly a more aggressive treatment strategy.

Management of right ventricular thrombi is challenging. The three therapeutic options for right heart thrombus are anticoagulation, thrombolysis or surgical thrombectomy. The optimal management is undocumented, since randomized controlled trials are lacking. Observational studies have reported comparable mortality for the three treatment options, 20–25 % after 14 days [14, 16, 17]. In contrast, a retrospective meta-analysis of 177 cases published between 1966 and 2000 the mortality was lower with thrombolysis (11 %) than with surgery (24 %) or anticoagulation (29 %) [18].

The European Cooperative Study on the clinical significance of right heart thrombi suggested a thrombus classification system for individualization of treatment [19]. Type A, extremely mobile thrombi in structurally normal hearts, are believed to originate in peripheral veins, and merely transit in the heart. Type B are immobile and though to originate within the right heart chambers, while type C thrombi elicit intermediate characteristics of both type A and B. While type A has been associated with high early mortality from massive pulmonary embolism and requires aggressive therapy, type B has a more benign course and can be managed with anticoagulation. In our patient with a type C thrombus who already had received six months of anticoagulation therapy, anticoagulation alone was not a therapeutic option, and thrombolysis of such a large thrombus carried high risk of fragmentation and further occlusion of pulmonary arteries. The patient was young with low operative risk, favoring surgical removal of the thrombus. Following surgery, long-term anticoagulation treatment was indicated to avoid new thromboembolic episodes. As the patient had experienced recurrent pulmonary embolism during anticoagulation therapy with warfarin, long-term anticoagulation treatment with low-molecular-weight heparin was initiated, as the treatment can be easily monitored by antiXa and the treatment effect is well documented for instance in cancer patients [20]. After 1 year, changing to warfarin in combination with aspirin will be considered [21, 22].

## Conclusion

The present case demonstrates the increased risk of severe thromboembolic complications following therapeutic splenectomy in hereditary spherocytosis, and the role of echocardiography and multimodality cardiac imaging in diagnosis of right ventricular thrombus as the cause of repeated pulmonary embolism.

### Consent

Written informed consent was obtained from the patient for publication of this Case report and any accompanying images. A copy of the written consent is available for review by the Editor-in-Chief of this journal.

## Additional files

Additional file 1: Transthoracic echocardiography showing the giant right ventricular thrombus in a parasternal short axis view. (WMV 5132 kb) Additional file 2: Contrast echocardiography in the parasternal short axis view, demonstrating the characteristic lack of contrast enhancement in the right ventricular thrombus. (WMV 5126 kb) Additional file 3: Cardiac magnetic resonance imaging in a sagittal plane demonstrating the piston-like shape of the thrombus, and how it protrudes through the pulmonary valve in systole. (WMV 1165 kb)

## Abbreviations

CT: computer tomography
MRI: magnetic resonance imaging
